# A Patient-Driven Mobile Health Innovation in Cystic Fibrosis Care: Comparative Cross-Case Study

**DOI:** 10.2196/50527

**Published:** 2024-07-31

**Authors:** Pamela Mazzocato, Jamie Linnea Luckhaus, Moa Malmqvist Castillo, Johan Burnett, Andreas Hager, Gabriela Oates, Carolina Wannheden, Carl Savage

**Affiliations:** 1 Department of Learning, Informatics, Management and Ethics Medical Management Centre Karolinska Institutet Stockholm Sweden; 2 Södertälje Hospital Södertälje Sweden; 3 Participatory e-Health and Health Data Department of Women’s and Child’s Health Uppsala University Uppsala Sweden; 4 Upstream Dream Bromma Sweden; 5 Pulmonary, Allergy and Critical Care Medicine The University of Alabama at Birmingham Birmingham, AL United States

**Keywords:** chronic illness, implementation, adoption, spread, patient-driven innovation, mHealth, mobile health, innovation, health care provider, motivation, interdependency, adaptability

## Abstract

**Background:**

Patient-driven innovation in health care is an emerging phenomenon with benefits for patients with chronic conditions, such as cystic fibrosis (CF). However, previous research has not examined what may facilitate or hinder the implementation of such innovations from the provider perspective.

**Objective:**

The aim of this study was to explain variations in the adoption of a patient-driven innovation among CF clinics.

**Methods:**

A comparative multiple-case study was conducted on the adoption of a patient-controlled app to support self-management and collaboration with health care professionals (HCPs). Data collection and analysis were guided by the nonadoption, abandonment, spread, scale-up, and sustainability and complexity assessment tool (NASSS-CAT) framework. Data included user activity levels of patients and qualitative interviews with staff at 9 clinics (n=8, 88.9%, in Sweden; n=1, 11.1%, in the United States). We calculated the maximum and mean percentage of active users at each clinic and performed statistical process control (SPC) analysis to explore how the user activity level changed over time. Qualitative data were subjected to content analysis and complexity analysis and used to generate process maps. All data were then triangulated in a cross-case analysis.

**Results:**

We found no evidence of nonadoption or clear abandonment of the app. Distinct patterns of innovation adoption were discernable based on the maximum end-user activity for each clinic, which we labeled as low (16%-23%), middle (25%-47%), or high (58%-95%) adoption. SPC charts illustrated that the introduction of new app features and research-related activity had a positive influence on user activity levels. Variation in adoption was associated with providers’ perceptions of care process complexity. A higher perceived complexity of the value proposition, adopter system, and organization was associated with lower adoption. In clinics that adopted the innovation early or those that relied on champions, user activity tended to plateau or decline, suggesting a negative impact on sustainability.

**Conclusions:**

For patient-driven innovations to be adopted and sustained in health care, understanding patient-provider interdependency and providers’ perspectives on what generates value is essential.

## Introduction

Patient-driven innovation in health care is an emerging phenomenon. An example of coproduction and *prosumerism* (where consumers produce what they consume), patient-driven innovations can be seen as the next logical step in health care evolution [1,2] and could fundamentally challenge the essence of what it means to be a professional health care provider. The number of publications about patient-driven or informal caregiver-driven innovations (ie, innovations that are both initiated and driven by patients or informal caregivers or both) mostly concern chronic conditions and have increased substantially in recent years [3]. However, the current literature does not examine the factors that influence the adoption, spread, and scale-up of patient-driven innovations in health care organizations [3]. The paucity of research studies evaluating and reporting the outcomes of patient-driven innovations has been suggested as a potential obstacle to their adoption in health care [4].

Cystic fibrosis (CF) is a complex chronic and genetic condition that affects respiratory and other organ systems [5]. Disease activity varies over time, and treatment requires a high degree of discipline and self-care outside of the clinical microsystem [6,7]. Patient-driven innovations in CF care have resulted in the development and dissemination of mobile health (mHealth) apps that support patients with CF and their caregivers in self-care and information sharing with health care providers [8-11]. Sharing of patient-generated health data has been associated with improved symptom control and quality of life and reduced health care use [12]. The COVID-19 pandemic has further demonstrated the value of and opportunity for patient-generated health data [13], as well as the importance of actively involving patients with CF and caregivers in critical conversations about care and self-care management [14].

However, implementing innovations in health care can be challenging [9,15-18]. The issue is even more pronounced if innovations are created or driven by patients [19], as that can challenge traditional hierarchical values and structures and professional identities. When implementation is not appropriately managed, mHealth apps fail to be adopted, are abandoned, or falter when they are scaled up or spread [20,21]. To increase the ability of hospitals, staff, and patients to adopt technological innovations, implementation approaches need to be anchored in the needs of patients and adapted to the organizational context and the wider system in which the new technology is implemented [21,22]. The level of organization and system complexity will influence the level of adoption by patients and providers [20,21,23-25].

Building on the field of complexity, we see complexity as a characteristic and property that emerges from 3 variables: the number of elements or components, often referred to as nodes (eg, actors and stakeholders); the number of interactions and interdependences between these nodes; and the variation within these nodes and interactions [26-28]. Successful implementation often requires a higher level of alignment between the purpose of the organization and its users [20,21,24]. The risk for failure increases with the level of complexity as health care systems respond to changes in unpredictable and nonlinear ways due to fuzzy organizational boundaries and interconnected actions with other actors that are often difficult to predict or even be aware of [21,29].

With patients and informal caregivers playing an increased role in health care, we need to know more about how their potential contributions can be realized through the adoption of patient-driven innovations in health care. Therefore, the aim of this study was to explain variations in the adoption of a patient-driven innovation among CF clinics. We posed the following research questions:

- How does the adoption of a patient-driven innovation, based on patterns of patient use, vary among clinics?
- What factors influence the level of adoption of the patient-driven innovation over time?

## Methods

### Study Design

This comparative multiple-case study used mixed (quantitative and qualitative) methods to explain differences in the adoption of a patient-driven innovation among patients with CF at 9 clinics. The Consolidated Criteria for Reporting Qualitative Research (COREQ) guideline [30] was followed in reporting this study (Multimedia Appendix 1). The study is part of the *Patient in the Driver’s Seat* research program conducted at the Medical Management Centre, Karolinska Institutet, Sweden. It is a 6-year program that studies how 5 patient-driven innovations are implemented in clinical practice and the daily lives of patients and their networks, 1 of which is the subject of this study.

### Theoretical Framework

This study was guided by the framework for theorizing and evaluating nonadoption, abandonment, and challenges in the scale-up (ie, building infrastructure to support full-scale implementation across an organization, locality, or health systems), spread (ie, replicating an intervention somewhere else), and sustainability of health and care technologies (nonadoption, abandonment, spread, scale-up, and sustainability [NASSS]) and the NASSS complexity assessment tool (NASSS-CAT) [29]. We chose the NASSS framework because it was designed to both analyze and prepare for the implementation of technology in health care. Its development process has been well described, lending surface validity to the framework, and it has garnered attention among researchers [20,31,32]. According to the framework, the dynamic interactions that influence the nonadoption, abandonment, scale-up, spread, and sustainability of technological innovations are inherent to the complexity within and between 7 domains:

- Condition: nature of the condition or illness, comorbidities, sociocultural factors
- Technology: material features, type of data generated, knowledge needed to use, supply model
- Value proposition: supply-side value for the developer and for the patient
- Adopters: staff, patients, caregivers
- Organization: capacity to innovate, readiness for technology, nature of adoption/funding decision, extent of change in team routines, work needed
- Wider context: political/policy, regulatory/legal, professional and sociocultural
- Embedding and adaptation over time: scope, organizational resilience [29]

Simple systems consist of a few components that interact in straightforward and predicable ways. Complicated systems have multiple components that interact in a predictable fashion. Complex systems have multiple and intricately related interactions that are constantly changing, unpredictable, nonlinear, and difficult to deconstruct [29]. NASSS and, practically, CAT can be used to distinguish between simple, complicated, and complex elements in the 7 domains [29]. The intention is to identify the multiple influences that are at play; to determine how complexity once identified can be reduced, addressed, or navigated; and to provide information and guidance to the involved actors on how to do so.

### The Patient-Driven Innovation

The technology (innovation) studied was a patient-controlled app (named *Genia*) that was originally designed as a patient-facing app to foster self-management. With the addition of a function to generate reports for providers, the app expanded its scope to become a patient-controlled information app for the coproduction of care that places the patient at the center of the decision-making process [10,11]. Founded in 2012 by a father of children with CF, Upstream Dream, which developed the patient-driven innovation, employs individuals with lived experience as patients or informal caregivers of a family member with a rare disease. The innovation was developed in collaboration with the Swedish CF community and upon research conducted at Karolinska Institutet and Dartmouth University. Launched in Sweden in 2015, it was introduced to all CF clinics in the country within a period of a few months. Subsequently, the innovation was piloted (2020-2021) and adopted (2021) as part of routine care in 1 pediatric CF program in the United States and is now the focus of a multicenter study. At launch, the patient-driven innovation was only compatible with the iOS platform. Android support was added later.

Upstream Dream is working to spread the innovation to other clinics in the United States and South America. The innovation has also been tested for use with other chronic medical conditions and has demonstrated improved patient engagement, patient-centered care, and practice-based learning, with the conclusion that the innovation can be recommended for other chronic conditions [31].

The main features of the patient-driven innovation are related to the tracking of symptoms and medications. What differentiates it from a regular personal health record for patients to record disease progression is that information can be shared with care providers, which is why the app was introduced through the clinics to reach patients with CF. Information that patients wish to share with their multidisciplinary clinical care team is submitted via previsit reports in the form of portable document format (PDF) files. Data are integrated into the Swedish National Cystic Fibrosis Quality Registry. In the United States, data are incorporated into the local electronic health record (EHR) [9]. Over time, additional features were added based on input from the clinics. These included an antibiotic use–reporting tool, “Antibiotic Check-in,” to support care and research on the use of new antibiotics and therapies; a medication-monitoring tool for the Orkambi medical treatment; and a “Health Check-in” feature to simplify communication of the information used by clinics for planning meetings and patient visits.

In Sweden, clinics were not charged to use the innovation, whereas in the United States, clinics paid an annual fee. The innovation was free for patients to use in both countries. Patients’ user activity can be seen as an outcome that reflects how well clinics integrate the app in their own care processes.

When the study was conducted, 2 generic health information and communication apps were provided by the regions and in use in the Swedish clinics, “1177” and “Always Open.” Neither app provides a comparable service (patient-controlled communication of disease activity) to the innovation; instead, all 3 apps provide complementary functions that did not interfere with one another and could therefore be used simultaneously. The 1177 app is a patient portal that provides information about illnesses and clinics, booking, and electronic medical records. The Always Open app (Swedish *Alltid Öppet*) was designed as a secure platform for providing remote care services, such as appointment reminders, prescription renewals, and video visits. Its availability was limited to 1 region, so only 2 clinics had access to it.

### Study Setting

The study was conducted at 1 CF center in the United States (referred to as clinic A) and all 8 pediatric and adult CF centers in Sweden (referred to as clinics B-I). All but 1 clinic had used the innovation for 7 years (2 years for the most recent clinic). The clinics were small, with 7-10 staff members (typically including pulmonologists, registered nurses (RNs), physiotherapists or respiratory therapists, psychologists or social workers, and dietitians), and focused specifically on CF. All were located at academic medical centers. One clinic in Sweden provided both pediatric and adult care, and the same staff served both patient populations. However, because adoption levels were different between these 2 patient groups, the data were presented separately.

Based on the inclusion of many different implementation settings, we expected different patterns of adoption. This would allow us to develop an empirical basis for both literal replication (cases that predict similar results) and theoretical replication (cases that predict different results for predictable reasons) [32]. The US site was selected because it was the first use case outside Sweden. We expected its inclusion to provide further insights into relevant contextual aspects.

### Data Collection

Quantitative data were collected to assess the level of adoption based on the number of active users (ie, patient activity). Since the innovation was designed to improve patient-provider communication, patient activity levels can be seen as an indicator of adoption. Anonymous user activity data for each clinic were extracted from Upstream Dream’s monthly reports on user activity for the period from March 2015 (when the innovation was implemented at the first clinic) to March 2022. At clinic A, where the innovation was implemented last, the user activity period was from February 2020 to March 2022.

Qualitative data were collected to understand how the innovation was perceived and used based on the NASSS domains and to identify specific interventions or factors that may have influenced user activity. A semistructured interview guide was developed in both Swedish and English (Multimedia Appendix 2). Questions were designed to elicit information about NASSS domains and their degrees of complexity [21], as well as to capture information about app integration into care processes. A precursor to the interview guide was first tested in a separate study [33] and revised based on that experience. Further input was sought from Upstream Dream to ensure that we would capture a holistic understanding of how the innovation was used in daily practice with respect to the different organizational contexts and potential differences in complexity. The interview guide was then piloted twice, with minor changes made to enhance the clarity of the questions in only the Swedish version.

Purposive and snowball sampling strategies were used to identify participants with knowledge of and personal experience with the innovation and who represented the professions involved in CF care. Upstream Dream’s clinical coordinators contacted the staff coordinators at each CF center on our behalf, who then connected us with the staff who expressed an interest to participate in the study. We contacted these individuals via email and followed up by telephone. In total, 21 participants were interviewed, including 2 key members of Upstream Dream, one of whom was interviewed twice (first, to develop a contextual understanding of the innovation and the supplier perspective and then to provide additional information and clarity after the adopter interviews), RNs, physicians, respiratory therapists or physiotherapists, a psychologist, a dietician, and a researcher involved in the development, implementation, and evaluation of the innovation in 1 of the clinics, without being a health care provider. This role distribution reflected typical staff distribution at the clinics. The CF centers involved had a small number of employees, which limited the number of potential participants. The number of participants was also limited by the high workloads brought on by the COVID-19 pandemic. Thus, the participants involved represented between 28% and 40% of working staff. Participants’ experiences with the innovation ranged from 3 months to 7 years.

Interviews were conducted online via the Zoom videoconferencing system due to the ongoing COVID-19 pandemic, in Swedish or English, and lasted 30-60 minutes. Interviews were conducted by 2 authors with training in qualitative research, with support from a senior researcher. The interviewers had no prior relationship with the participants. Audio was digitally recorded and transcribed verbatim.

### Data Analysis

Quantitative data on user activity were analyzed using descriptive statistics. We calculated the percentage of active end users per month among the total number of patients and the percentage of active users on average over the entire period. Active users were defined as end users who logged on and used some of the basic app features within a 6-month period. As clinic A first introduced the patient-driven innovation as a pilot study, the percentage of active users for that period was calculated based on the pilot sample size and after the end of the pilot was calculated based on the clinic size. The descriptive statistics (ie, maximum end-user activity and average over the entire period) provided insights into the level of adoption. We defined low (<25%), middle (25-50%), and high (>50%) activity levels based on the maximum percentage of end-user level achieved.

To put user activity data into an organizational context, we complemented the descriptive data with statistical process control (SPC) charts. SPC charts were created for each clinic to identify whether and when statistically significant changes in app adoption levels occurred and whether these were sustained. SPC makes it possible to determine whether a change is a matter of chance (ie, common-cause variation) or due to a specific happening or intervention (ie, special-cause variation) [34-36]. We used P charts with the following rules to identify special-cause variation: rule 1/3 sigma violation (1 point +/– the upper control limit/lower control limit [UCL/LCL], with the control limits set to +/–3 sigma), rule 2/shift (8 successive consecutive points above or below the centerline), rule 3/trend (6 or more consecutive points steadily increasing or decreasing), and rule 5/hugging the centerline (15 or more consecutive points within +/–1 sigma on either side of the centerline) [37].

Qualitative data were analyzed using content analysis (directed and inductive) [38], process mapping, and complexity analysis. Interview transcripts were read through repeatedly to develop familiarity and then subjected to directed qualitative content analysis [39]. Two authors together identified meaning units, which they abstracted to condensed meaning units and added as “sticky notes” to the MIRO online whiteboard for visual collaboration, where they were directed to 1 of the NASSS domains (ie, categories). The condensed meaning units were labeled with descriptive codes. Where the codes did not fit the framework, additional subcategories were created through traditional inductive content analysis [39]. All codes and the categorization were reviewed and corroborated by 2 other authors. During the analysis, interview data from suppliers and HCPs were kept separate, and the former were used solely to provide contextual information about specific interventions and factors that were integrated in the SPC charts (eg, timeline of interventions that could have influenced user activity).

To support the cross-case comparison, the traditional approach to reporting qualitative analyses was then transformed into tabulated form based on the original NASSS framework and expanded to include the additional subcategories.

Process maps were created for each clinic based on interview data to illustrate how the innovation was integrated into clinical work processes. The process maps and the qualitative analysis were shared with participants in an informant validation process. Five clinics made small adjustments. The suppliers provided additional corroborating feedback, where needed, for the clinics that did not respond.

We diagnosed complexity levels (see the definitions of simple, complicated, complex in the theoretical framework) of the NASSS domains for each clinic by analyzing interview data using the NASSS complexity table (Multimedia Appendix 3) and the NASSS-CAT Short survey, which was specifically designed to assess and differentiate between complexity levels [29]. Both analyses were combined to generate the complexity assessment. Although the survey was originally intended to spark a reflective discussion, we used the reflective discussion created in the interview setting to diagnose the level of complexity. For the qualitative analyses and the complexity assessment, conflicting interpretations were discussed until consensus was reached.

Qualitative data were first analyzed case by case but then triangulated with quantitative data to develop initial explanations of variation in adoption. These were then tested against the data and the analyses to identify those domains or interacting domains that could explain the observed patterns. In a process akin to modified analytic induction [38], when falsifying evidence was found, the explanatory model was dropped. This process involved vigorous discussion and iterative cycles to narrow and refine the explanatory models that are presented in the cross-case comparison.

### Ethical Considerations

Ethical approval was obtained from the Swedish Ethical Review Authority (approval number 2019-03849). The study followed the Swedish Research Council’s ethical principles for humanities and social science research. Participants were informed orally and in writing about their rights and what study participation would entail. Written and verbal consent was obtained from all participants prior to commencement of interviews. Quantitative data were completely anonymous. Qualitative data were pseudonymized and deidentified prior to coding and analysis. In presenting the findings, we made efforts to maintain participants’ privacy and confidentiality, referencing HCPs only with their pseudonym identifiers and clinic letters (eg, “HCP01, clinic A”). No participant received any compensation for participating in the study.

## Results

In this section, we present the triangulation of the qualitative and quantitative data first with user activity levels, and then a cross-case comparison based on the complexity assessment.

### Participant Details

In total, 21 participants (n=16, 76.2%, women and n=5, 23.8%, men) were interviewed. Of the 21 participants, 9 (42.9%) were RNs, 5 (23.8%) physicians, 4 (19%) respiratory therapists or physiotherapists, 1 (4.8%) psychologist, 1 (4.8%) dietician, and 1 (4.8%) researcher.

In 2 of the clinics (D and H), we were able to interview only 1 (4.8%) participant each; the remaining 7 clinics were represented by 2 (9.5%) or more participants: clinic A, n=3 (14.3%); clinic B, n=4 (19%); clinic C, n=4 (19%); clinic E, n=2 (9.5%); clinic F, n=2 (9.5%); clinic G, n=2 (9.5%); and clinic I, n=2 (9.5%).

### User Activity Level

The end-user activity levels showed that the innovation was adopted by patients at all clinics; there was no evidence of nonadoption. Based on the maximum end-user level achieved, we found 3 clusters: high-adoption clinics A (maximum n=40, 65%), B (maximum n=60, 58%), and C (maximum n=66, 96%); medium-adoption clinics D (maximum n=125, 35%), E (maximum n=30, 47%), and F (maximum n=60, 25%); and low-adoption clinics G (maximum n=120, 19%), H (maximum n=116, 23%), and I (maximum n=155, 16%).

**Figure 1:**
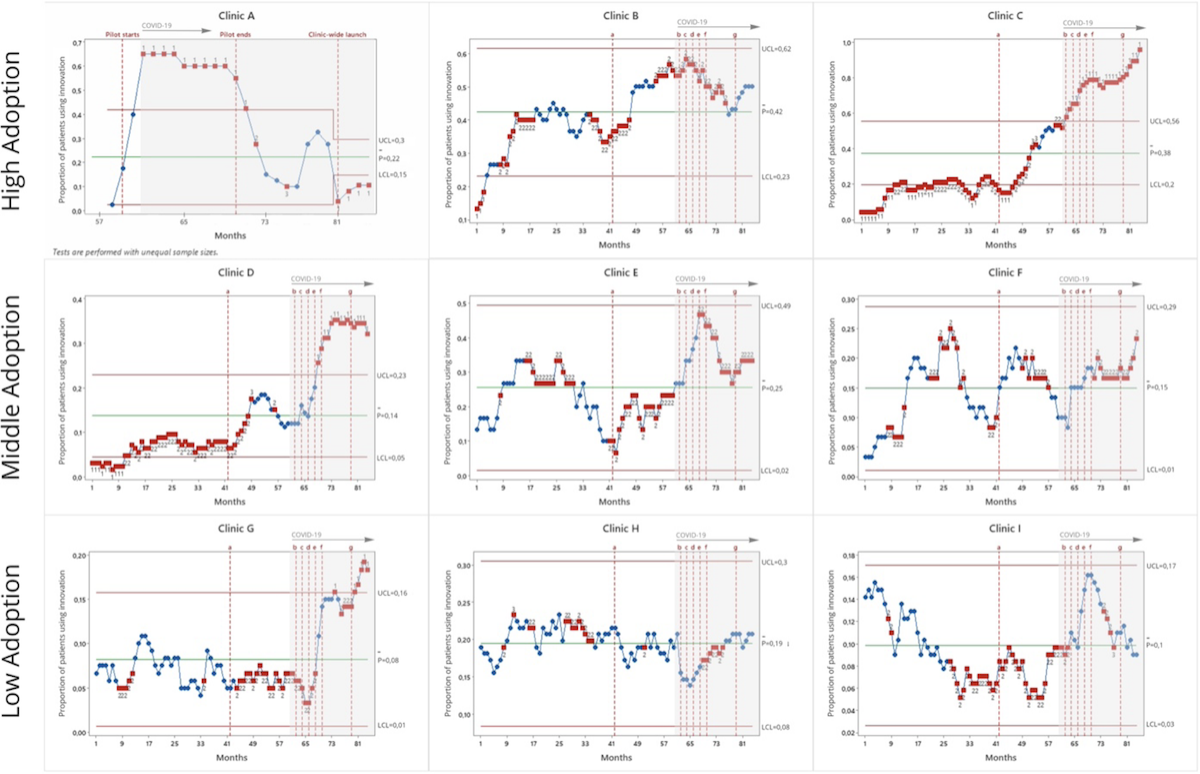
SPC analysis of end-user activity at the different clinics organized as high (top row), middle (middle row), and low (lower row) end-user adoption. The UCL and LCL were defined as +/– 3 SDs from the centerline [37]. The y-axis uses different scales to be able to better discern the trends over time. The blue circles represent common cause variation, whereas the red squares represent special cause variation, with numbers indicated according to SPC rules. LCL: lower control limit; SPC: statistical process control; UCL: upper control limit.

To further explore user activity levels over time, SPC charts were created (Figure 1) for each clinic to plot the user activity level over 81 months. In the SPC charts, specific interventions or events retrieved through the interviews, which may have influenced user activity levels over time, are indicated as vertical dotted lines and labeled as a-g. These included the introduction of new features, research-related activity, and the COVID-19 pandemic.

New features included the launch of Orkambi medication monitoring (intervention a) in month 42, which likely explains the increased use among 5 clinics (B-F), particularly among pediatric clinics. Antibiotic Check-in was launched in Swedish clinics in month 62 (intervention b), which was followed by a campaign (intervention d), which could explain the increased activity in 7 (87.5%) of the 8 Swedish clinics (C-I). The introduction of an Android-compatible version in month 68 (intervention e) opened the innovation up to all patients and caregivers with a smartphone or tablet. Health Check-in (intervention f) may have contributed to the observable increase around month 70 (clinics C, D, F, and H).

The influence of research-related activities was mainly identified in clinic A, in which there was a rapid increase in activity starting month 60, which plateaued. This corresponded to when the innovation was first introduced in clinic A as a year-long single-group pilot study (n=40 participants, pre-post design) [40]. When the patient quota for the pilot was met (first vertical dotted line, Figure 1), 40% of the invited patients were active users. Activity declined after the pilot ended but began to rise again (month 81) when the decision was made to launch the innovation clinic-wide. The consistency of this increase continued after the last point recorded in Figure 1 until activity was halted again in preparation for a new study (data not included in the SPC). Clinic C launched a digitization research project (intervention c), which required participants to use the innovation, and later launched a second digitization project (intervention g) in month 79.

The COVID-19 pandemic also appeared to influence user activity levels. Clinic A partially transitioned to telehealth visits during the pandemic, which made the Health Check-in feature desirable as patients could upload photos and other information. This may have contributed to increased user activity. In contrast, for clinics E, F, H, and I, a deterioration (months 70-78) was concomitant with an active choice not to focus on the innovation due to strained resources.

### Cross-Case Comparison: Complexity Assessment Linked to Adoption Level

**Figure 2:**
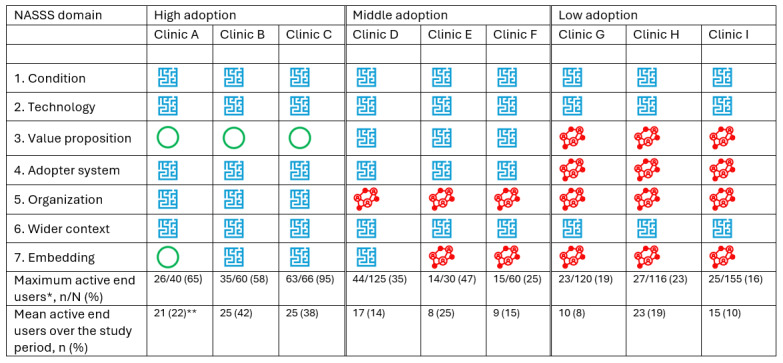
Complexity assessment for NASSS domains grouped by adoption level based on maximum end-user activity. Green circles represent simple, blue labyrinths complicated, and red network symbols complex. *Minimum activity levels were not reported because data collection started when there were 0 active users for all clinics. **For clinic A, n was calculated based on active users over the pilot study. NASSS: nonadoption, abandonment, spread, scale-up, and sustainability.

Specific characteristics of the clinics related to each NASSS domain gleaned from the interviews and the process maps are presented in Multimedia Appendix 4. In terms of complexity, all clinics viewed the nature of the *condition*, *technology*, and *wider context* domains as complicated (Figure 2). Differences were found in the *value proposition*, *adopter system*, *organization*, and *embedding* domains. The cross-case comparison presented next was organized around levels of adoption, integrating data from the SPC analysis and exploring differences and similarities in the NASSS domains.

#### High-Adoption Cluster (Clinics A-C)

The first group perceived the value proposition as simple, the adopter system and organization as complicated, and embedding as either simple or complicated. The staff had a shared perception that there is a clear benefit to using the innovation (ie, the innovation-facilitated meeting of patient and provider creates value in health care for this chronic condition). This was aligned with their view that value in health care for this chronic condition is created in the patient-provider meeting. Although going through reports lengthened the previsit planning process, the innovation was perceived to save time through more concise patient visits.

Just doing that, [opening reports in weekly team meetings] will change it a little bit, it is going to lengthen our [team] meeting a bit (…) So it helps everybody to be prepared ahead of time and hopefully make the visit more concise.

HCP14, clinic A

For clinics B and C, where Antibiotic Check-in was used, these data were highly valued for research purposes and research was seen as an integral component of high quality care. For clinic C, this was corroborated by SPC analysis (intervention c).

The adopter system was viewed by clinics A-C as complicated. Patient-controlled data were viewed as integral to previsit planning, suggesting a view of patients as active contributors to the cocreation of care and of the staff as having a responsibility to ensure that patients contributed with these data.

Way more attention is being paid now to this patient-generated data rather than the core clinical data. I mean, we still look at lung function and microbiology and what not, but patient-reported symptoms and outcomes are a bigger part of the discussion now, as well as patient preferences and…and goals and what people want to focus on. So that is 1 change that has happened.

HCP15, clinic A

This person-centered culture was reflected in the organization domain, which the staff perceived as complicated, as organizational routines and care pathways needed to be flexible to reinforce the importance of patient input and the use of the innovation as an integral source of information for previsit planning. For example, if a patient had either not downloaded the innovation or submitted a report, clinics A and C had routines to complete those tasks together with the patient (Multimedia Appendix 4). Even though these clinics saw a clear value in the innovation, they still expressed the difficulties of cultural change and that it requires key drivers.

We often do not have the personnel for this. It needs to be carried out, in addition to the usual work. And there has to be someone driven to be able to push these questions forward.

HCP01, clinic C

Embedding was seen as simple or complicated based on how mature the routines for incorporating user information (the reports) into the patient pathway were. In clinic B, the continual increase in adoption observed in Figure 1 could be explained by the clinic’s routines of using the innovation with the whole clinical team, as well as its regular communication with the developer. For example, clinic A had clear plans and used collective reflection among the staff for embedding the technology in the short and the long term.

The good thing is that the way we have set up this pragmatic trial is that they are [clinicians] not required to do anything extra than they normally do, so they are not required to log in on a separate platform or a dashboard or anything extra. Everything is embedded and integrated, which makes it an easier sell.

HCP15, clinic A

Clinics A-C saw a match between what they valued (the patient-provider interaction) and the value proposition of the innovation because it improved the quality and efficiency of the patient-provider encounter. These clinics had existing and further developed their routines to ensure and reinforce patient use of the innovation. Moreover, they behaved as if they “co-owned” the innovation either by conducting research studies or by taking responsibility for patient training. In the adopter system, most of the staff had clearly defined roles in relation to the use of the innovation, as well as established routines for group reflection. Participants valued patients’ role in the cocreation of care.

#### Middle-Adoption Cluster (Clinics D-F)

The second group perceived the value proposition and adopter system as complicated and organization and embedding as complex. The value proposition was seen as complicated because the technology’s desirability was contested, and the business case for adoption was deemed unclear. The participants experienced a mismatch between the condition and technology that reflected itself in their view of the value proposition: clinics D and F (adult clinics) believed that the technology better serves pediatric patients, who typically receive outpatient care and need to report symptoms, whereas adult patients with CF in Sweden are often hospitalized and their symptomatology easier to track. The staff felt that the graphical user interface is less appropriate for adults.

Regarding the adopter system, clinics E and F questioned the appropriateness of care providers in a public institution to convince their patients to use a product from a private company.

If a patient is completely on top of it with their treatment and medications and everything, then it feels a little like a car salesman if I am to try and sell something that is not a directive of the hospital. And that role we all feel is a bit annoying. But if you can show a direct benefit to using [the innovation], then it feels good.

HCP12, clinic F

Organization was seen as complex because of difficulties integrating the innovation in the workflow. For example, patient-generated reports were scheduled on the weekly agenda at clinics E and F but seldom discussed. Work routines also included contact with the supplier, who felt that clinic D demonstrated heightened commitment. This was mirrored in the SPC data, which showed a clear increase in adoption toward the end of the data collection period. However, patient workflow processes were not established. Adoption for all 3 clinics was largely the responsibility of individual clinicians and, due to individual levels of enthusiasm or work practices, gave rise to variation (Figure 1). This impacted patients.

We are a center where not everyone is on the team in the same way. I think that is a factor for whether, depending on which doctor one meets, there will be a question about [the innovation] or not (…) It becomes person dependent in a crazy way.

HCP19, clinic E

It impacted staff as well.

If everyone did it, there would not be any extra work, but since we cannot manage to get everyone here doing it, I end up trying to take the main responsibility (…) Sometimes I sit and go through [the reports] (…) So, I have a bit of extra work, but it is too difficult to create a routine for such a thing if not all patients use it, then it can become forgotten.

HCP18, clinic H

In clinics E and F, adoption relied heavily on champions, which could explain the decrease observed in months 26-43 (Figure 1), which was also compounded by a severe staff shortage. Embedding was deemed complex at clinics E and F as they were forced to prioritize resources due to the COVID-19 pandemic, which shifted focus away from the innovation and was reflected in the user activity decrease. Moreover, they felt the questions prior to the introduction of Antibiotic Check-in and Health Check-in were too generic.

#### Low-Adoption Cluster (Clinics G-I)

The third group perceived the value proposition, adopter system, organization, and embedding as complex. Like clinics D-E, these providers had a negative experience of advocating for a privately owned app. They were among the first to adopt the innovation, at a point where app features were minimal and the app was limited to 1 platform. They felt that this effort to get patients to use an underdeveloped app negatively impacted the patient-provider relationship. The SPC analysis for clinics H and I (Figure 1) confirmed that early adopters struggled to maintain adoption as the user activity level either remained stable (clinic H) or decreased (clinic I). The addition of new features, although potentially increasing the value of the innovation, was offset by previous experiences, which had worn them out.

It is now actually that you should start trying to get patients to use [the innovation]. But in [this region], we have sort of worn ourselves out because we already did it 4-5 years ago.

HCP18, clinic H

The innovation was seen as undesirable by most of the staff at these clinics, and they perceived their patients were equally uninterested. Several providers mentioned that patients do not want to “have their illness in an app,” as symptom tracking can become a reminder of how sick one has been and add yet another task, when patients with CF are already “drowning in health care” (HCP06). Some also felt adults have a hard time changing their ways. These opinions demonstrated a mismatch between the value of the technology and the needs and challenges related to the condition.

Adopter systems were deemed complex because, although at all clinics the staff had to learn new skills, the staff at clinics G-I felt the innovation poses a threat to their professional identity and scope of practice and felt patients find the innovation challenging. Clinicians preferred digital technologies available from the regions, rather than from a private company. Here, they referred to 1177 and Always Open as examples of such tools, which they also perceived served a clearer purpose. Clinicians were skeptical of how the app was introduced and that it did not come from within the clinic.

It was not we as providers who went to an app developer and said, “We want a tool.” Rather, it was they who came from the outside and said, “You need a tool, and here it is.”

HCP03, clinic G

Clinicians at pediatric clinics believed there is a particular need for medication tracking among adult patients, whereas the staff at adult clinics expressed the opposite. Past experiences with other technological interventions and how well they were received also influenced how hesitant or open clinics were to innovation.

Organizations also demonstrated complexity; none of clinics G-I had integrated the innovation into their clinical workflow, and the innovation was used on an individual rather than on a team basis, which put pressure on the single user (Multimedia Appendix 3). This was reinforced by the special cause variation observable in clinic G (consecutive points below/above the center line, months 69-83), where the activity dipped down before shooting up and could be linked to a champion staff member who led the adoption but left temporarily before returning. The clinics saw funding as a barrier to implementing new technologies, including the innovation. Clinic I mentioned a severe resource pressure, including hiring stops (frozen posts), which halted the use of the innovation, especially under the stress of the pandemic. The embedding system was complex due to the clinic’s inability to adapt the innovation use to critical and unforeseen events (eg, the COVID-19 pandemic).

## Discussion

### Principal Findings

In this study, the adoption of a patient-driven innovation was studied using a complexity-based framework and tools for the introduction of technology in health care, NASSS-CAT. The innovation was developed as a patient-controlled information app to support the self-management of CF and communicate disease related–activity with health care providers. Although we found no evidence of nonadoption or clear abandonment of the app, distinct patterns of innovation adoption were discernable based on user activity data (ie, low, medium, and high adoption). The perceived value proposition of the technology and the experienced complexity were associated with different levels of adoption. Research activity and the introduction of new app features positively impacted adoption. In clinics that adopted the innovation early or those that relied on champions, user activity tended to plateau or decline, suggesting a negative impact on sustainability.

#### Perceived Complexity Influencing Adoption

There was little variation between clinics regarding perceptions of the condition and technology domains. Differences in complexity were seen within the value proposition, adopter system, organization, and embedding and adaptation domains. The more complex these domains were perceived to be, the lower the level of adoption was.

The perceptions of value identified in this study demonstrate a patient-provider interdependency (ie, both the provider and the patient must value and use the patient-driven innovation, or else it will lead to a downward spiral of abandonment). As Floch et al [8] found, “Self-management enfolds a collaboration between patients and [health care providers].” This suggests that studying providers’ experiences of using patient-driven innovations can be an important perspective as patients’ and providers’ behaviors are 2 sides of the proverbial same coin. One could expect that a patient-driven innovation, as an example of prosumerism, would entail a higher level of acceptance or be more highly valued than an innovation external to the clinical context.

All clinics seemed to agree that there is a clear need to focus clinic visits on what patients value. Where the value proposition was perceived as simple, HCPs saw the innovation as a solution, or at least worth testing as a solution. Although going through reports lengthened weekly previsit planning, the staff saw that the innovation enables shorter and more concise patient visits [9]. This mirrors the findings for another patient-centered care app for patients with CF [8]. Clinics with lower adoption described the innovation as interfering with the patient-provider interaction, since they believed they already knew their patients well due to the chronic nature of CF and perceived the app as an affront to their professionalism. The time providers spent with their patients to elicit this information was seen as a demonstration of how they valued their patients. The difference between the 2 interpretations of value could be paraphrased, from the perspective of the professional, as “we value our patients’ time” versus “we value our time with patients.” In the former, providers focus on what matters to the patient with the help of the innovation; in the latter, providers try to find out what is the matter with the patient through a person-to-person conversation without the innovation and the information it provides.

In terms of working with the adopter system and organization, our findings suggest a need to work with the context to integrate a new patient-driven innovation in health care. In this respect, despite prosumerism, this patient-driven innovation is not different from other innovations in health care [33]. Working with a broader group of adopters, not just champions, and integrating the patient-driven innovation with care processes appeared to facilitate adoption. If context is not addressed by suppliers, there is a risk that the perceived value of the innovation will be influenced by the perceived complexity of the setting. This lived experience of complexity could not be explained in terms of differences in the medical condition or technology. Instead, it appeared to be more dependent on how care processes had evolved and the human (in)ability to *deal* with variation, uncertainty, complexity, and ambiguity in everyday work life: complexity was in the eye of the beholder.

#### Co-ownership and Trust

When the app was first launched, the company took responsibility for staff and patient education, with the intention of having as little disturbance as possible in the clinics. However, clinics that took shared responsibility for the patient-driven rollout had higher and sustained adoption levels. As studies in behavioral economics have demonstrated [41], a higher level of perceived co-ownership leads to a higher evaluation of the object in question. Co-ownership invites the staff to learn and understand more about the patient-driven innovation, which could explain why merely relying on champions can be associated with lower adoption and responsibility for patient training and research with higher adoption.

Most clinics raised questions about the financial motives of the company behind the patient-driven innovation. Two clinics questioned whether it is appropriate for them as medical providers to “sell” a product from a private company. Particularly, during the early rollout, the staff felt that pushing a premature version of the innovation on patients is a violation of their professional integrity. The distrust of a private business overshadowed the patient-driven prosumer nature of the innovation that should have engendered trust. This distrust could be related to a commonly held negative view of privatization in Sweden or may indicate that the company’s patient origins had not been communicated clearly.

#### Utility of NASSS-CAT

This study used NASSS-CAT as a framework and tool to analyze the implementation of a patient-driven innovation. We found it helpful to characterize single domains but less suitable to explore the interaction between domains. The framework is innovation centric, which risks generating a bias that values innovation per se regardless of its suitability for addressing the challenge at hand or the purpose of the hosting clinic or organization.

Another issue relates to the essence of the complexity captured with CAT. Our findings support the basic tenet of NASSS-CAT that adoption is inversely related to the level of complexity. However, a closer look into the data suggests that what was captured may have been individuals’ perceptions of complexity related to lived experiences rather than the actual contextual complexity related to the level of interdependency. This corroborates preliminary observations of the original CAT [29]. Thus, results may be more indicative of the maturity of the complexity mindset of individuals [42] rather than the contextual complexity itself. To develop adoption strategies based on such data would be tantamount to developing treatment strategies based on an incorrect diagnosis.

A more accurate assessment of contextual complexity could be achieved by exploring the level of agreement between understanding the challenge and the proposed response [25,26,43]. These 2 questions are simpler to ask and evaluate to generate actionable data: less agreement would suggest a higher degree of complexity and therefore a need for strategies that facilitate learning [43,44]. More direct implementation works when things are simple (ie, greater agreement).

### Strengths and Limitations

Directed content analysis inherently has some biases due to the use of a preselected theory [39]. However, several measures were taken to mitigate this limitation. For example, we used open-ended questions in the interview guide, and multiple authors were involved in all the steps of the qualitative analysis.

There was variation in the number of participants per clinic, which could have influenced the analysis. Clinics where the innovation was perceived more positively and used to a greater degree also tended to yield more interviews. This difference may reflect both resource availability and the perceived value of the innovation. Overall, the number of interview participants was limited by the number of employees at each clinic and the COVID-19 pandemic. The literature suggests that theoretical saturation is usually attained at around 12 interviews [45]. This exceeds the number of staff members working with the innovation in most of the clinics we studied. Despite our small sample size, the participants’ specific knowledge about the innovation and the care processes in each clinic contributed to strengthening information power [46]. Moreover, the triangulation of qualitative and quantitative data strengthened the trustworthiness of the findings.

Not all clinics provided feedback on the process maps, although all were given the opportunity. To further improve trustworthiness, process maps were checked again against the transcripts and reviewed by the developer’s clinic coordinator, who had insight into the clinics and staff.

The total number of potential users was limited because the innovation was first released in an iOS version only. As we did not have data on the proportion of potential users who had an iOS smartphone, we may have overestimated the number of potential users, in particular prior to the release of the Android version.

This study did not examine patient outcomes related to the innovation or satisfaction with the innovation. Interviewing patients and informal caregivers would add valuable perspectives to that of providers.

### Conclusion

Patient-driven innovations could be highly relevant for health care, but their adoption has seldom been explored from the perspective of health care providers. We found that providers play a significant role in the adoption of patient-driven innovations in health care: patients cannot do it alone. Health care providers who make an effort to reduce the perceived complexity in the adoption process, simplify their processes, take co-ownership of the innovation, and work on its adoption and improvement as a team, rather than relying on change champions, improve their capability to support the adoption and sustainability of innovative ideas developed by patients. For patient-driven innovations to be adopted and sustained in health care, understanding patient-provider interdependency and providers’ perspectives on what generates value is essential.

## Appendix

Multimedia Appendix 1 Consolidated Criteria for Reporting Qualitative Research (COREQ) checklist.

Multimedia Appendix 2 Interview guide.

Multimedia Appendix 3 Nonadoption, abandonment, spread, scale-up, and sustainability (NASSS) domain areas and case-specific descriptions.

Multimedia Appendix 4 Process maps.

## Abbreviations

CAT: complexity assessment tool
CF: cystic fibrosis
HCP: health care professional
LCL: lower control limit
mHealth: mobile health
NASSS: nonadoption, abandonment, spread, scale-up, and sustainability
RN: registered nurse
SPC: statistical process control
UCL: upper control limit

## Data Availability

No additional data are available. The data sets generated and analyzed during this study are available from the corresponding author upon reasonable request.
