# Supplementary figures and images for: A Patient-Driven Mobile Health Innovation in Cystic Fibrosis Care: Comparative Cross-Case Study
Source: J Med Internet Res. 2024 Jul 31;26:e50527. doi: 10.2196/50527 (PMC11325108; doi:10.2196/50527)

Appendix 1: COREQ

ff
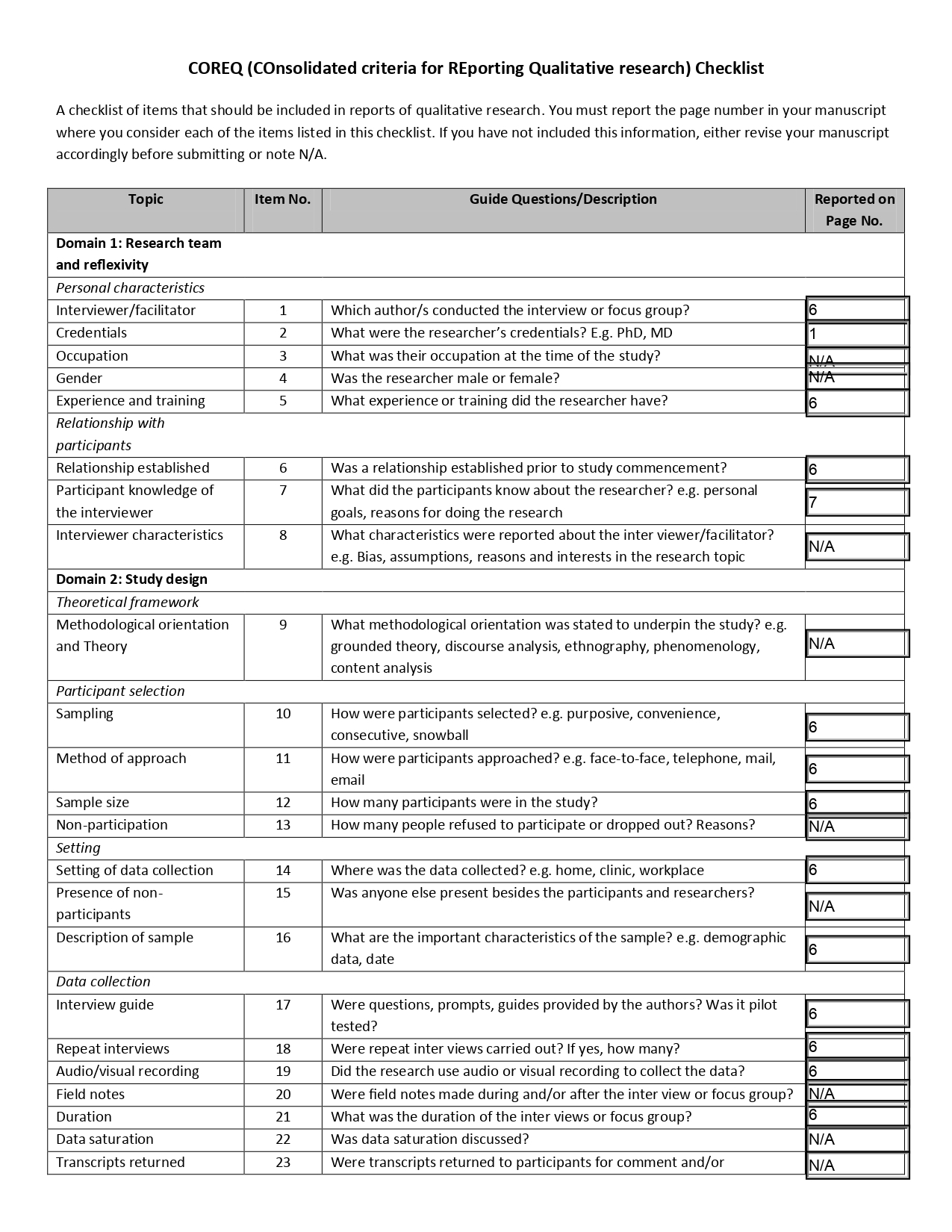


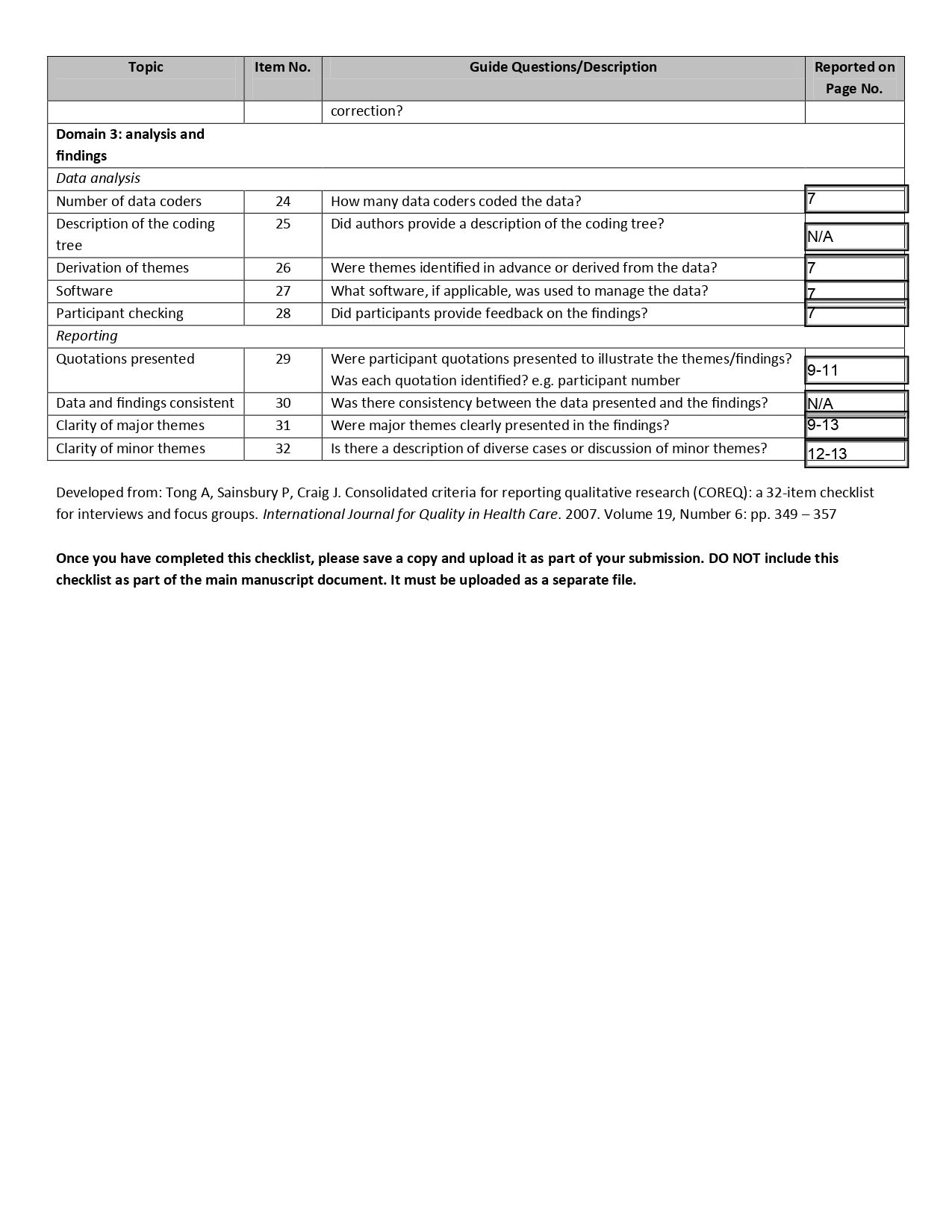

Supplement: Multimedia Appendix 1 [file jmir_v26i1e50527_app1.docx]

## Appendix 3: Process maps

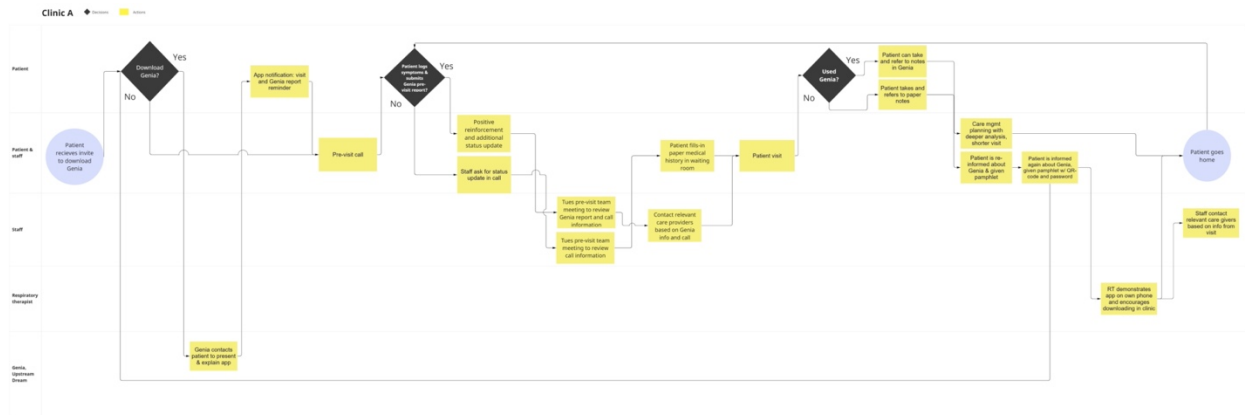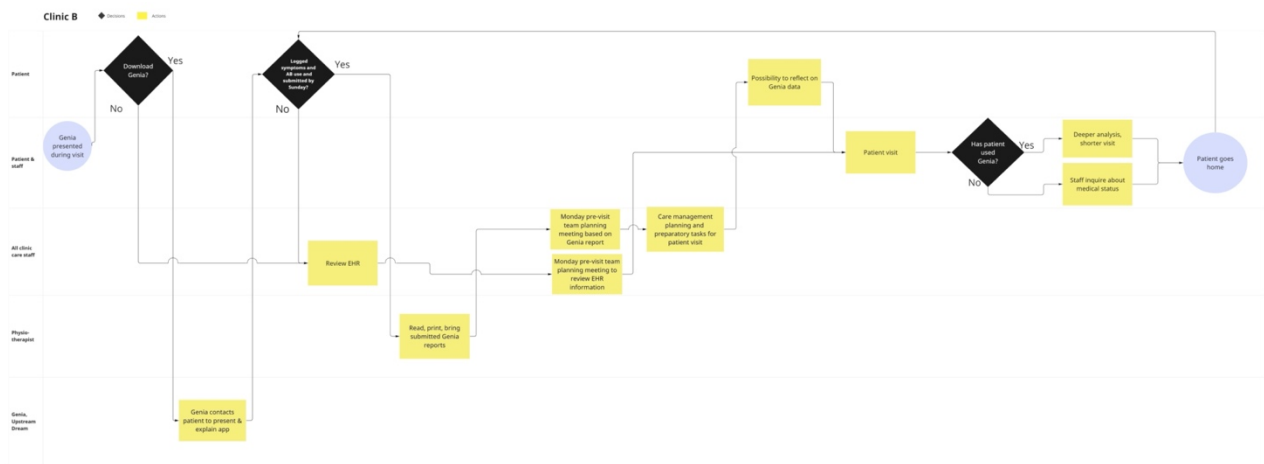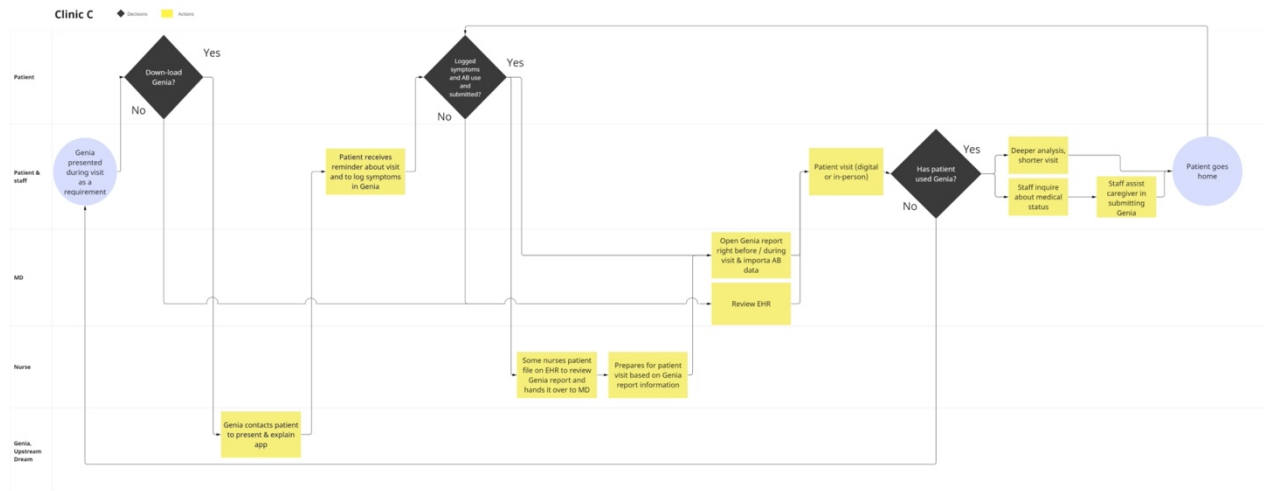

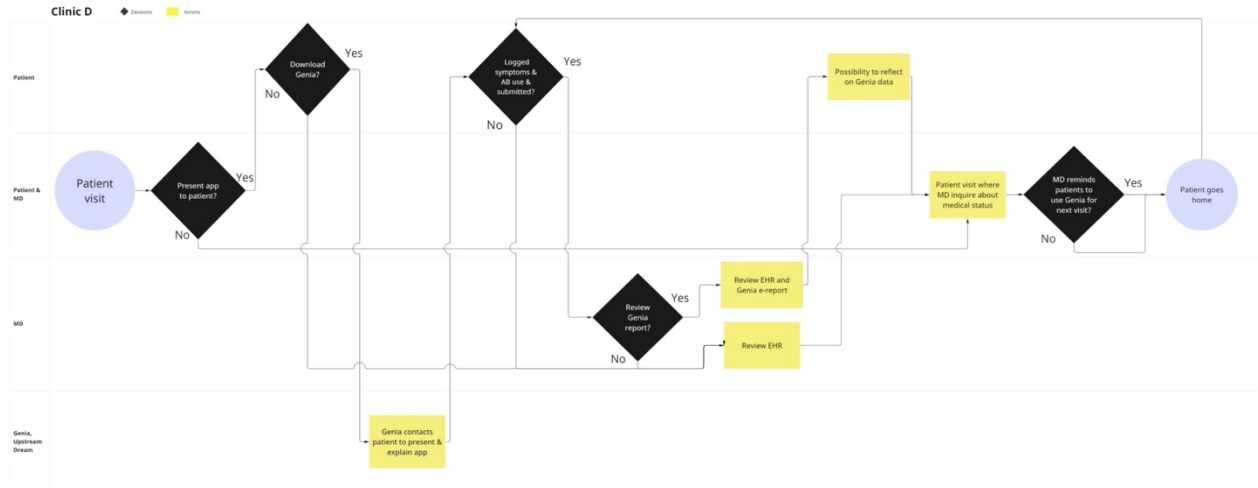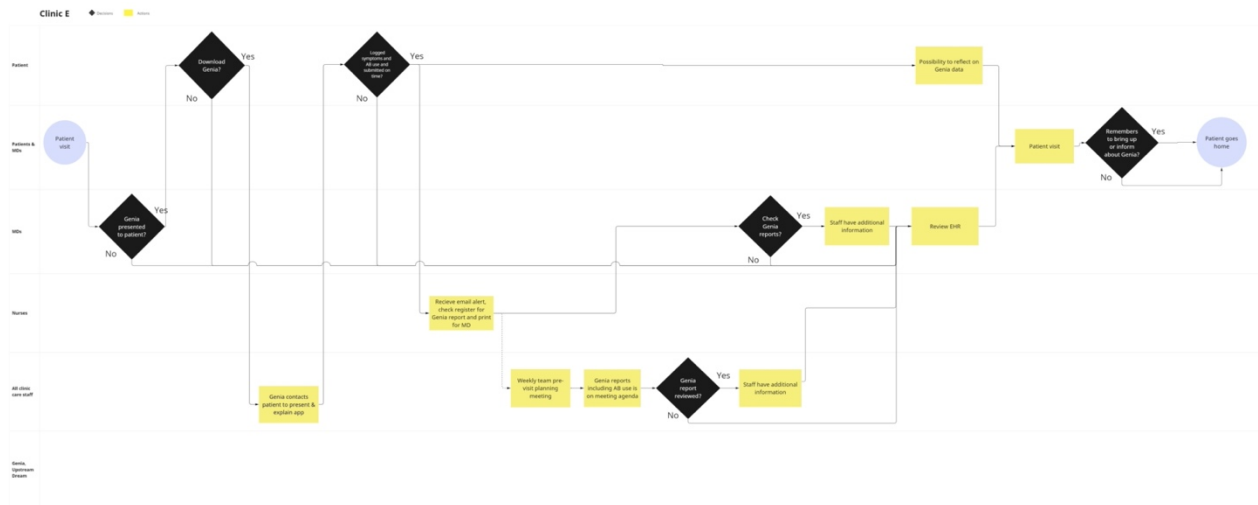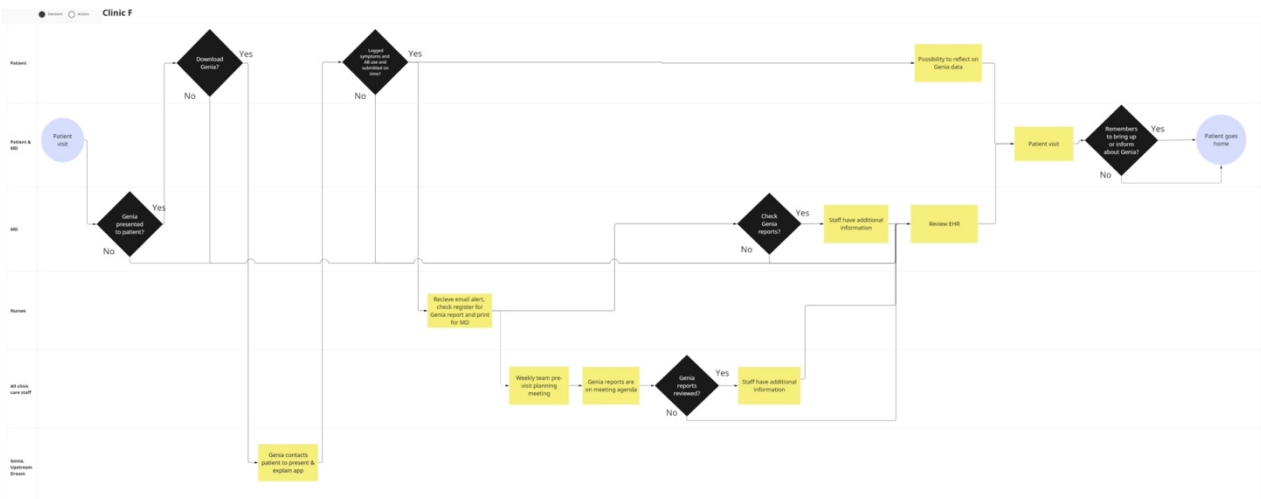

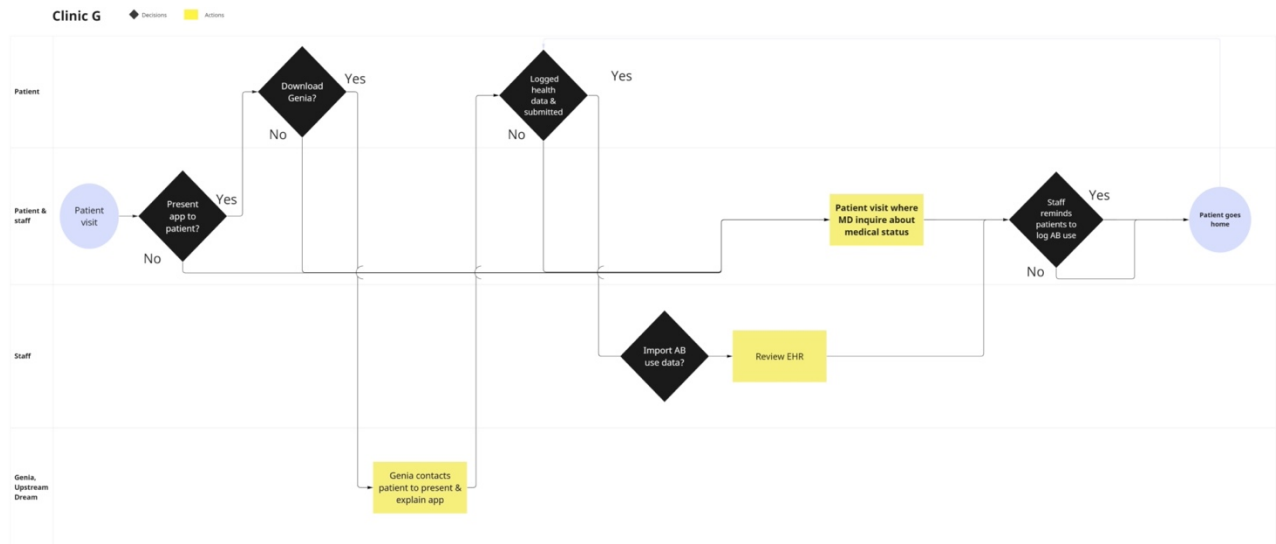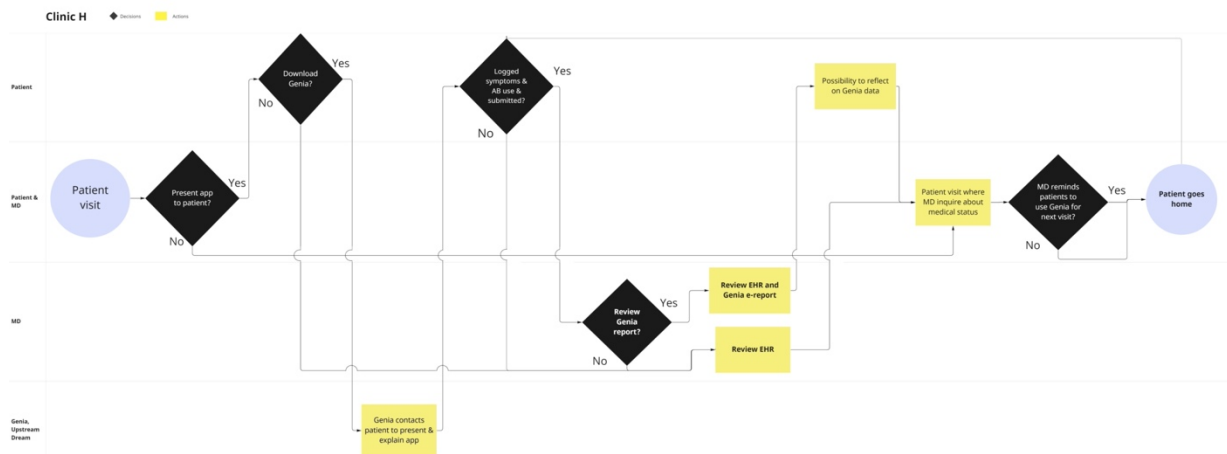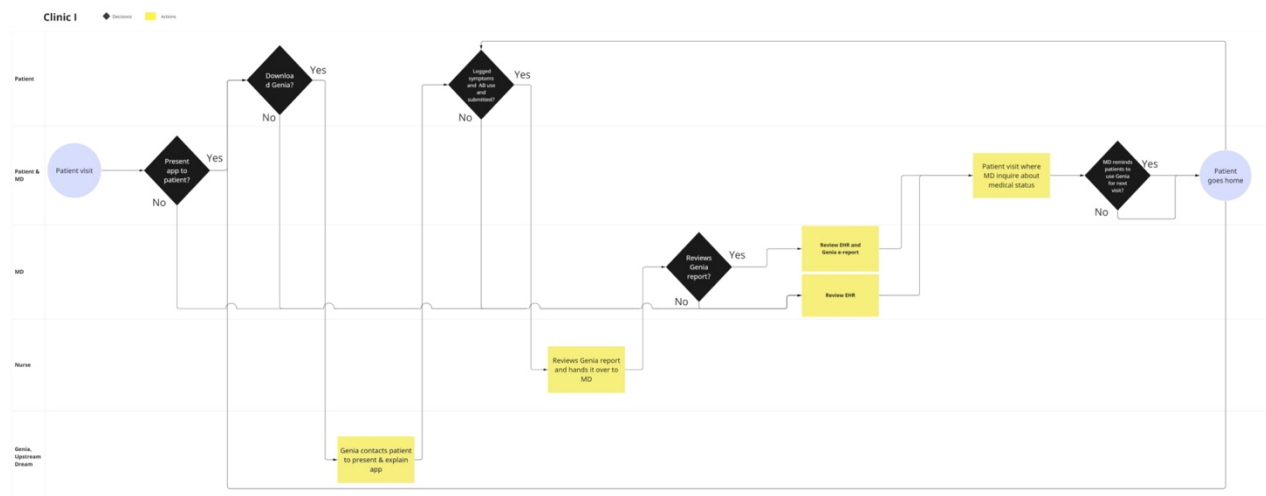

Supplement: Multimedia Appendix 4 [file jmir_v26i1e50527_app4.pdf]
