# Supplementary material for: A Patient-Driven Mobile Health Innovation in Cystic Fibrosis Care: Comparative Cross-Case Study
Source: J Med Internet Res. 2024 Jul 31;26:e50527. doi: 10.2196/50527 (PMC11325108; doi:10.2196/50527)
Supplement: Multimedia Appendix 2 [file jmir_v26i1e50527_app2.docx]

Interview guide for health care professionals

The **aim** of these interviews is to capture your experiences and usage of Genia, as well as to explore differences in how Genia is used and integrated among different clinics and professionals. Please speak freely under the interview, which will take maximum one hour.

**Are you okay with the interview being recorded?** *(Start recording)*

Just to get it on tape, you have consented to the interview being recorded. Before we get started, your participation is optional, and you can withdraw at any point. Everything said under the interview will be treated confidentially and your answers will remain anonymous.

**Do you have any thoughts or questions before we begin? I DO NOT WORK FOR [THE COMPANY]**

# About you (Condition)

1. What led you to work with patients with cystic fibrosis?
   1. Please describe what it is like to work with this patient group.
   2. What would you say are the biggest challenges to working with CF patients, and what is most rewarding?

# Value Proposition

1. Do you feel that Genia addresses any of the aforementioned challenges? Or that it could?
2. What did you expect when you first heard of Genia? Did it go as expected?

# Technology

1. How is Genia used by you and your patients?
   1. In preparing for an appointment (pre-visit **Reports**)
   2. To structure an appointment/during the appointment (reports, **health check-in**, **antibiotic check-in**) note-taking (caregivers)
   3. Communication (the patient’s network and reporting to HCS)
   4. In-between appointments (the patients fill out the health check, antibiotic check-in, observations)
   5. How do you access the data?
2. Does Genia impact how you deliver care?

# Adopter system

1. What changes in staff roles, practices, and identities are implied by the implementation of Genia? *(Threat to professional identity, values, or scope of practice; risk of job loss)*
   - 1. Initial concern that patient-generated data would impact work flow/process - and now?
     2. **clinical management protocol** developed to provide clear process e.g. bring up reports in weekly pre-visit meetings
2. What is expected of the patient (and/or immediate caregiver) *(eg, log on, enter data, converse, initiate change sin therapy, make judgments, organize)*
   1. and is this achievable by, and acceptable to, them?
3. What is assumed about the ex-tended network of lay caregivers? (Family members, friends…)

# Organization

1. How open to taking on new workways and/or technologies would you describe the health care community as? (in your state and/or nationally)
2. How ready did you feel that you and your team were when you began using Genia as a part of your work routine?
3. What is needed to begin using Genia?
   1. What support did you receive? (training) article mentions 1-day face-to-face training for CF center. Any since? New staff? Sufficient?
   2. How much change to your work routines was required?
   3. How have you as a team worked to implement Genia?
4. Have you as an organization done any evaluation of Genia? If so, how and why?

# Wider Context

1. How does Genia impact the way you and your patients coordinate their healthcare with other health care centers? E.g. social worker, mental health counselor
2. Do you see any possible ripple effects from implementation Genia?
   1. Effects on the profession, politics, legal aspects, collaboration...

- HIPPA – challenge?
- US: 2^nd^ most spoken is Spanish; 3^rd^ language Chinese – problem with Eng app?
- In development stage: Respiratory check-in, etc,

# Adaptation/Embedding over time

1. In conclusion, what would you say that you and the clinic have learned through using Genia as a part of your work?
   1. Have there been any defining moments for you or the organization in this process?

# Concluding

1. Regarding everything we just discussed, is there anything that you would like to add or ask?

Thank you so much for taking the time to participate in an interview.

ASK ABOUT OTHERS IN THE TEAM
