# Supplementary material for: A Patient-Driven Mobile Health Innovation in Cystic Fibrosis Care: Comparative Cross-Case Study
Source: J Med Internet Res. 2024 Jul 31;26:e50527. doi: 10.2196/50527 (PMC11325108; doi:10.2196/50527)
Supplement: Multimedia Appendix 3 [file jmir_v26i1e50527_app3.pdf]

Appendix 2: NASSS domain area and case specific description

0 = not mentioned in interview/not applicable, 1 = agree, 2= disagree. PDI=patient-driven innovation

NASSS domain area and case-specific description	Response by clinic								
	A	B	C	D	E	F	G	H	I
1 CONDITION									
1A Nature of condition is life-long, multi-systemic, and time consuming	1	1	1	1	1	1	1	1	1
1A Patients need to become increasingly active in their treatment with age	0	1	1	0	0	0	0	0	0
1B Socioeconomic factors such as language, educational and economic status, and living conditions, need to be factored into the care plan.	1	1	1	1	1	1	1	1	1
2 TECHNOLOGY									
2A Experienced technical difficulties at launch	2	1	1	0	0	0	0	0	0
2A Questions were too generic at launch	2	0	1	0	1	1	0	0	0
2A Patients expected data to submit automatically.	1	1	1	0	1	1	1	1	1
2A Became more user-friendly with updates.	1	1	1	1	1	1	1	1	1
2B Provides a useful complementary picture of the patient's life e.g. activities, mental health, and appetite	1	1	1	2	2	2	2	2	1
2B Data from reports used by all clinical disciplines e.g. dieticians in team meetings.	1	1	2	2	1	1	2	2	2
2D Staff feel the PDI is appropriately focused for their patient target age group	1	1	1	1	1	2	2	2	2
2D PDI developed by a private company, questions raised about financial motivation	2	2	2	0	1	1	1	1	1
2D PDI developed by a private company, questions raised about its sustainability	1	1	1	0	0	0	0	0	0
2D Prefer to use existing publicly funded tools (with less functionality) instead of the PDI	2	2	2	2	2	2	1	1	1
3 VALUE PROPOSITION									
3B Pre-visit reports aided providers in preparing for a patient visit	1	1	1	2	1	1	2	2	2
3B PDI served as a reminder for providers and patients of what to cover during visits	1	0	0	0	0	0	0	0	0
3B ACI was the most desired feature	2	1	1	1	1	1	1	1	1
3B Improved pre-visit planning was a value generated by the PDI	1	1	0	0	1	1	2	2	2
3B Provider use was important for reinforcing patient use	1	1	1	0	1	1	0	0	1
3B Other mHealth tools and/or traditional communication routes such as email work fine	1	0	0	1	0	0	0	1	0
3B There is no "one-size-fits-all" and there is a need for more tailored options for specific patient segments	1	2	2	2	2	1	1	1	1
3B More data could make work more difficult for staff	2	2	2	2	2	2	1	1	1
4 ADOPTER SYSTEM									
4A The designated group that handles PDI-related tasks desires more activity and interest from other professions	2	2	2	2	2	2	2	1	1
4A Individual/champion bears the onus of responsibility for the implementation and use of the PDI	2	2	2	2	1	1	2	1	2

4A Nurses bear the onus of responsibility for using the PDI	2	2	2	2	2	2	2	2	1
4A Physicians reported lack of time to check PDI reports	2	2	2	2	1	1	2	2	1
4A Reports are printed and read as a paper copy	2	1	2	2	2	2	2	2	2
4A Found it difficult to incorporate the PDI into clinical routines.	2	2	2	2	2	2	1	1	1
4A Questioned whether it is appropriate for medical provider to "sell" a product from a private company	2	2	2	2	1	1	1	0	0
4B Did not see the PDI as an additional task asked of informal caregivers	1	1	1	0	1	0	0	1	0
4A Provider takes responsibility for patient education	1	1	1	2	1	2	2	2	2
4A Require the Antibiotic Check-in of patients	2	0	1	2	2	2	2	2	2
4B Emphasized the difficulty for older patients to change their routines as a reason for not using the PDI	0	0	0	0	1	1	1	0	1
5 ORGANIZATION									
5A Faced severe resource pressure and understaffing e.g. frozen posts	2	2	2	0	1	1	0	0	1
5B Piloting the PDI in a research study within one's own context was a key factor for successful implementation	1	0	1	2	2	2	2	2	2
5B Research on the PDI piqued interest	1	1	1						
5B Staff questioned PDI's clinical and practical relevance	2	2	2	2	2	2	1	1	1
5D Specific professional group took main onus of responsibility for PDI-related tasks	2	1	2	1	2	2	2	1	1
5D Team involves all HCPs (clinical professions) in PDI routines	1	1	1	2	2	2	2	2	2
5D Clinic holds weekly pre-visit planning team meetings	1	1	2	2	1	1	2	2	2
5D PDI compliments/digitalizes pre-existing pre-visit planning routines	1	1	2	2	1	1	2	2	2
5D Have integrated the PDI into clinical workflow	1	1	1	2	2	2	2	2	2
5D Has integrated the PDI into clinical workflow in theory, but high practical variation – on agenda but dependent on who among staff	2	2	2	2	1	1	2	2	2
5D Have integrated the PDI into clinical workflow but variation among MD use	2	2	2	2	2	2	2	2	1
5D PDI is not used by staff	2	2	2	2	2	2	1	2	2
5D Process maps: Sequential simple (clear routines)	1	1	1	2	2	2	2	2	2
5D Process maps: Iterative complicated (variation in routines)	2	2	2	1	2	2	1	2	2
5D Process maps: Complicated	2	2	2	2	1	1	2	1	1
5E Process maps: Clear standardized value adding process	1	1	2	2	2	2	2	2	2
5E Process maps: Planned standardized value adding process but not practiced	2	2	2	2	1	1	2	2	2
5E Process maps: Non-standardized value adding process	2	2	1	2	2	2	2	2	2
6 WIDER CONTEXT									
6A Valued the ACI as a data source which, if used more by patients, could serve research purposes and could contribute to reduced antibiotic-resistance, overall antibiotic use, and eventual cost savings.	2	1	1	0	1	1	1	0	1
6A Felt the PDI strengthened collaborations with other CF centers	1	1	1	0	1	1	1	1	1
6A PDI could be used across specialty clinics as they share the same EHR.	1	2	2	2	2	2	2	2	2
6A Expressed concerns over data privacy	1	1	2	2	2	2	2	1	2
7 EMBEDDING AND SCALE-UP									
7A Currently working on spreading the PDI to other clinics internationally	1	2	2	2	2	2	2	2	2

7A Saw Covid-19 pandemic as an opportunity to digitalize health care.	1	2	1	2	2	2	2	2	2
7B COVID-19 pandemic forced reprioritization of resources and focus away from the PDI	1	2	2	2	1	1	2	1	2
